# Atypical interseasonal respiratory syncytial virus infection in hospitalized children up to 12 years old

**DOI:** 10.1111/irv.13183

**Published:** 2023-08-09

**Authors:** Gabriela Barbosa, Ana Helena Perosa, Nancy Bellei

**Affiliations:** ^1^ Escola Paulista de Medicina, Laboratório de Virologia Clínica Universidade Federal de São Paulo (UNIFESP) São Paulo SP Brazil

**Keywords:** acute respiratory infection, hospitalized children, respiratory syncytial virus

Dear editor,

Respiratory syncytial virus (RSV) is the major cause of acute lower respiratory infections (ALRI) associated to hospitalization in children. In 2019, it was estimated that RSV was related to over 100 000 deaths in children aged 0 to 60 months.^1^ RSV outbreaks usually occur during fall and winter seasons. In the Southern Hemisphere, RSV cases peak usually in March and April, corresponding to the beginning of the fall season.^2^ During the COVID‐19 pandemic, the pattern of circulation of the respiratory viruses was importantly impacted due to measures to contain the spread of SARS‐CoV‐2.^3^ In this study, we aimed to assess the RSV‐related hospitalization in children in the period of 2022–2023 in Hospital São Paulo, Brazil.

This observational cohort study was conducted in compliance with institutional guidelines, approved by the Ethics Committee of Universidade Federal de São Paulo (CEP/UNIFESP n. 4.753.851). Data were obtained from the records of the Clinical Virology Laboratory, in Hospital São Paulo.

We investigated all children aged 0 to 12 years old hospitalized presenting respiratory symptoms. For every individual, a nasopharyngeal or a tracheal aspirate was collected. A real‐time polymerase chain reaction (RT‐PCR) was performed to detect RSV infection in the samples; primers and probes are described elsewhere.^4^ Positive samples were also submitted to RT‐PCR to detect subtypes RSV‐A and RSB‐B.^5^ Descriptive statistics and Fischer's exact test were analyzed in GraphPad 9.5.

This analysis included 566 hospitalized children from January 2022 to May 2023 aged 0 to 144 months (median 24 months; SD: 41.16; IQR: 7–60), 261 girls (46.2%) and 305 boys (53.8%). The overall infection rate for RSV was 12.3% (70/566). The age group 0 to 6 months represented 28.5% (37/130) of positive cases, with the highest infection in 2022–2023, as expected (*p* < 0.0001). The age group 7 to 11 months represented 11.7% (7/60) of infections, followed by 12 to 23 months 11% (8/73). Age group 24 to 59 months had 8.3% (11/135) and children >60 months presented 4.2% (7/168) of RSV infection. Except for the month of August, the detection of RSV cases was possible throughout the year of 2022. In 2023, most cases occurred in April. The distribution of cases per age group and period are in Figure 1.

**FIGURE 1:**
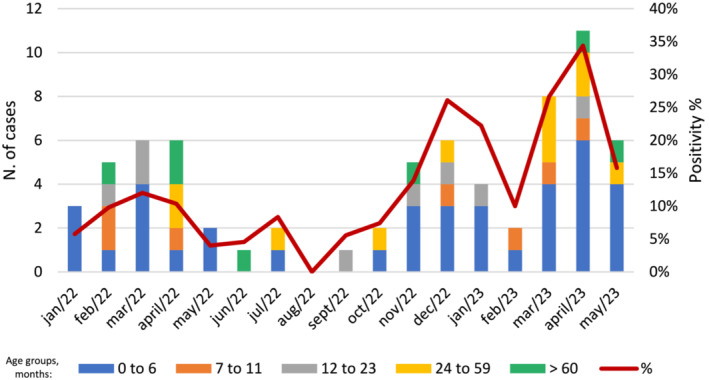
RSV‐related hospitalization in children aged 0 to 12 years old per month from January 2022 to May 2023.

We then attempted to verify the subtype associated with the infections. Of the 70 positive samples, 63 were subtyped, in which 49.2% were identified as RSV‐A and 50.7% as RSV‐B. In 2022, RSV‐B was predominant (74.3%), whereas in 2023, 81.5% of cases were identified as RSV‐A. At least one comorbidity was reported by 51.2% (290/566) of the included children, and 27.1% (19/70) among RSV positive cases. Twelve children under 24 months old presented comorbidities 63.2% (12/19).

Our findings reflect the atypical circulation of RSV among hospitalized children during the analyzed period. Moreover, we observed important rates of infection in children older than 24 months, resulting in RSV‐related hospitalization in a group of less impact, as demonstrated in another study conducted in the Southern Hemisphere.^6^ An interesting shift of circulation of RSV‐A and RSV‐B was evidenced in 2022 and 2023, even though the real impact regarding severity of the disease of the two serotype remains unclear.^7^

This present study highlights that it is crucial to keep the surveillance of RSV infections, particularly in children older than 2 years old with comorbidities; this alongside with an interseason RSV testing could provide a better comprehension of the impact of RSV circulation, seasonality, and association with an increase of hospitalization and disease severity.

## AUTHOR CONTRIBUTIONS

**Gabriela Barbosa:** Data curation; formal analysis; software; validation; writing—original draft. **Ana Helena Perosa:** Data curation; formal analysis; investigation; methodology. **Nancy Bellei:** Conceptualization; funding acquisition; project administration; resources; visualization; writing—review and editing.

## CONFLICT OF INTEREST STATEMENT

The authors have no conflicts of interest to declare.

### PEER REVIEW

The peer review history for this article is available at https://www.webofscience.com/api/gateway/wos/peer-review/10.1111/irv.13183.

## ETHICS STATEMENT

This study was conducted in compliance with institutional guidelines, approved by the National Commission for Research Ethics (CEP/UNIFESP n. 4.753.851).

## Data Availability

The data that support the findings of this study are available upon reasonable request from the corresponding author. The data are not publicly available due to privacy or ethical restrictions.
